# RNA Biomarker Trends across Type I and Type II Aerobic Methanotrophs in Response to Methane Oxidation Rates and Transcriptome Response to Short-Term Methane and Oxygen Limitation in Methylomicrobium album BG8

**DOI:** 10.1128/spectrum.00003-22

**Published:** 2022-06-09

**Authors:** Egidio F. Tentori, Shania Fang, Ruth E. Richardson

**Affiliations:** a School of Civil and Environmental Engineering, Cornell University, Ithaca, New York, USA; University of Minnesota

**Keywords:** methanotrophs, methane oxidation, *Methylomicrobium album* BG8, *Methylocystis parvus* OBBP, RNA-seq, *pmoA*, biomarkers

## Abstract

Methanotrophs, which help regulate atmospheric levels of methane, are active in diverse natural and man-made environments. This range of habitats and the feast-famine cycles seen by many environmental methanotrophs suggest that methanotrophs dynamically mediate rates of methane oxidation. Global methane budgets require ways to account for this variability in time and space. Functional gene biomarker transcripts are increasingly studied to inform the dynamics of diverse biogeochemical cycles. Previously, per-cell transcript levels of the methane oxidation biomarker *pmoA* were found to vary quantitatively with respect to methane oxidation rates in the model aerobic methanotroph Methylosinus trichosporium OB3b. In the present study, these trends were explored for two additional aerobic methanotroph pure cultures grown in membrane bioreactors, Methylocystis parvus OBBP and Methylomicrobium album BG8. At steady-state conditions, per-cell *pmoA* mRNA transcript levels strongly correlated with per-cell methane oxidation across the three methanotrophs across many orders of magnitude of activity (*R*^2^ = 0.91). The inclusion of both type I and type II aerobic methanotrophs suggests a universal trend between *in situ* activity level and *pmoA* RNA biomarker levels which can aid in improving estimates of both subsurface and atmospheric methane. Additionally, genome-wide expression data (obtained by transcriptome sequencing [RNA-seq]) were used to explore transcriptomic responses of steady-state *M. album* BG8 cultures to short-term CH_4_ and O_2_ limitation. These limitations induced regulation of genes involved in central carbon metabolism (including carbon storage), cell motility, and stress response.

**IMPORTANCE** Methanotrophs are naturally occurring microorganisms capable of oxidizing methane, having an impact on global net methane emissions. Additionally, they have also gained interest for their biotechnological applications in single-cell protein production, biofuels, and bioplastics. Having better ways of measuring methanotroph activity and understanding how methanotrophs respond to changing conditions is imperative for both optimization in controlled-growth applications and understanding *in situ* methane oxidation rates. In this study, we explored the applicability of methane oxidation biomarkers as a universal indicator of methanotrophic activity and explored methanotroph transcriptomic response to short-term changes in substrate availability. Our results contribute to better understanding the activity of aerobic methanotrophs, their core metabolic pathways, and their stress responses.

## INTRODUCTION

Methanotrophs are microbes capable of getting all their carbon and energy from methane, the second most abundant greenhouse gas after carbon dioxide (1, 2). Methanotrophs represent the only known biological methane sink and play a pivotal role in the global methane cycle (3). Methanotroph-based biotechnologies, coupling methane oxidation with production of value-added products like methanol, biopolymers, single-cell proteins, lipids, and enzymes, have been gaining interest (4–6). Therefore, they represent a possible lynchpin in biorefinery scenarios for conversion of methane and/or biogas to bioproducts.

Methanotrophic bacteria are found in the phyla *Proteobacteria* and, more recently, *Verrucomicrobia* (7, 8) and NC10 (9). The most widely studied methanotrophs are from the classes *Gammaproteobacteria* and *Alphaproteobacteria*, and based on physiological characteristics, they were historically divided into type I and type II methanotrophs, respectively (10, 11). However, methanotrophs can exhibit considerable metabolic flexibility even among methanotrophs of the same type. For example, they are capable of using nitrate or ammonium as a nitrogen source (12, 13), nitrogen fixation (14), carbon accumulation under nutrient-limited conditions (15, 16), and long-term survival under starvation conditions (17–21).

A defining characteristic shared by almost all methanotrophic bacteria is methane monooxygenase (MMO) enzymes, which initiate methane oxidation (22). Methane is converted to methanol, followed by formaldehyde, which can be either incorporated as biomass or ultimately converted to CO_2_. Two distinct MMOs can be found in methanotrophs, particulate methane monooxygenase (pMMO) and soluble methane monooxygenase (sMMO). Methanotrophs that possess both MMOs express pMMO under high copper-to-biomass ratios and sMMO at low copper-to-biomass ratios (23). Gene and gene expression amounts of pMMO and sMMO subunits, genes *pmoA* and *mmoX*, respectively, are used to quantify populations of methanotrophs, their activities, and their transcriptional and phenotypic response to changes in conditions (24–26). As nearly all aerobic methanotrophs possess pMMO, gene *pmoA* is the preferred biomarker to determine both their abundance and methane oxidation activity.

Approaches that quantify both biomarker mRNA transcripts and/or enzymes in addition to microbial amounts have been suggested as estimators of microbial contributions in biogeochemically relevant processes (27). The utility of protein and mRNA biomarkers in microbial communities has been demonstrated previously, in both identifying microbial function and determining *in situ* activities of specific community members (28–31). Periods of temporal variations affecting microbial activity would also be reflected by changes in their respective mRNA or enzyme pools.

In methanotrophs, increases in *pmoA* gene copies and *pmoA* transcript levels correlate with increased methane oxidation (6, 32–34) and have been proposed as quantitative indicators of their activity (35, 36). Recently, a strong correlation between steady-state per-cell *pmoA* transcript levels and per-cell methane oxidation rate was demonstrated in the type I methanotroph Methylosinus trichosporium OB3b grown in membrane bioreactors (37). If similar relationships between biomarker amounts and activity are shared across different methanotrophs, they could allow dynamic inference of *in situ* methane oxidation rates.

The goal of this study was to explore correlations between methane oxidation biomarker amounts and methanotrophic activity across aerobic methanotrophs species and obtain further insight into their transcriptomic response to dynamic conditions of substrate availability. To assess this, aerobic methanotrophs Methylocystis parvus OBBP (type II) and Methylomicrobium album BG8 (type I) were grown in membrane bioreactors under different conditions where biomarker *pmoA* gene and transcript amounts (via quantitative PCR [qPCR] and reverse transcription-qPCR) were measured. Additionally, effects of short-term (less than one retention time [RT]) substrate (O_2_ and CH_4_) limitation and recovery on RNA biomarker expression were explored in *M. album* BG8 using a targeted qPCR approach and RNA sequencing to determine temporal expression patterns under dynamic conditions influenced by environmental factors.

## RESULTS AND DISCUSSION

### Steady-state reactor performance.

**FIG 1:**
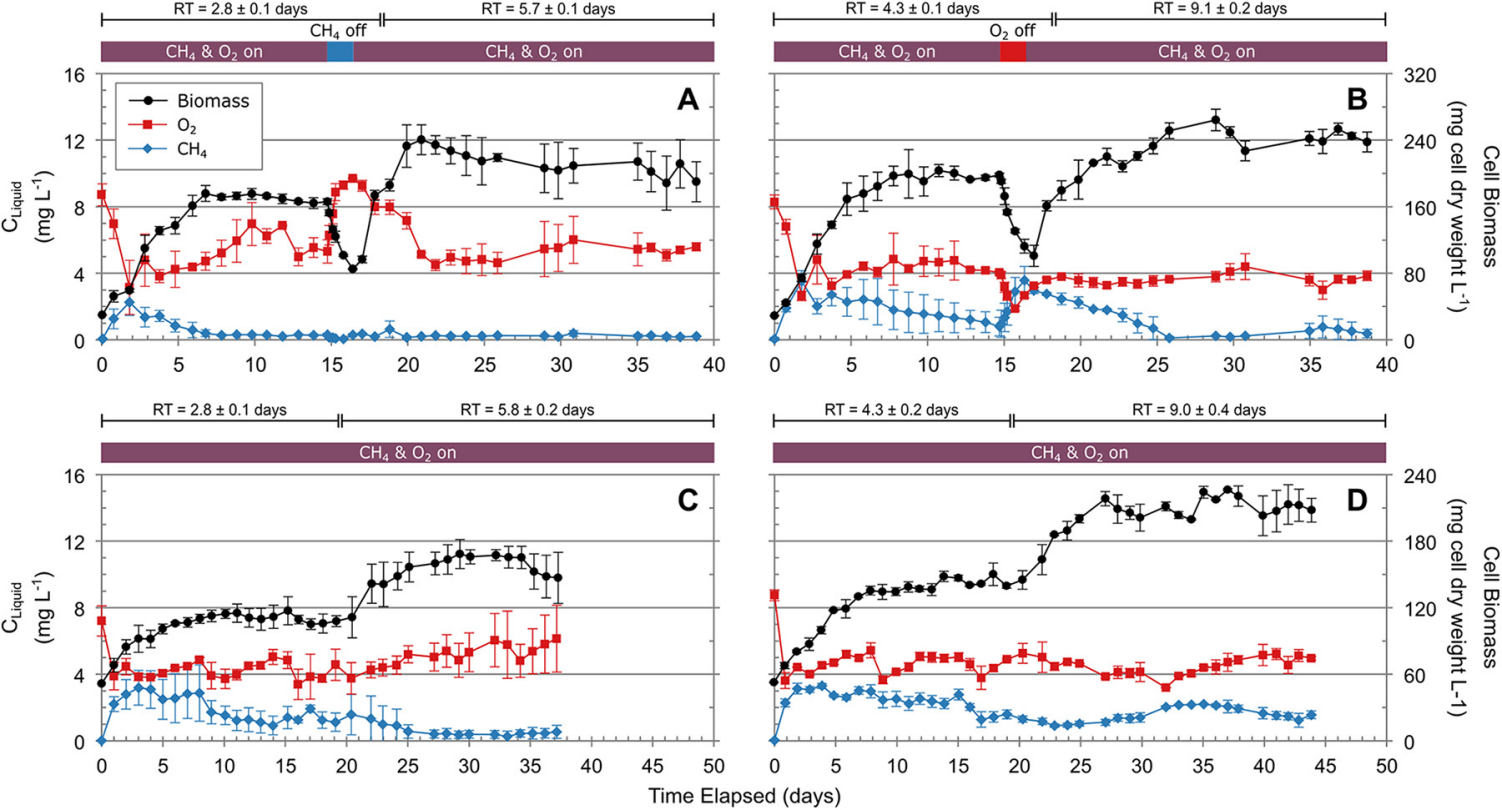
Performance of *M. album* BG8 and *M. parvus* OBBP reactors. (A) *M. album* BG8, 2.8-day RT, CH_4_ limitation, transition to 5.7-day RT; (B) *M. album* BG8, 4.3-day RT, O_2_ limitation, transition to 9.1-day RT; (C) *M. parvus* OBBP, 2.8-day RT, 5.8-day RT; (D) *M. parvus* OBBP, 4.3-day RT, 9.0-day RT. Aqueous concentrations of CH_4_ and O_2_, left *y* axis, and biomass, right *y* axis. Data are means ± standard deviations from triplicate reactors.

Reactor flow rates were monitored, and retention times (RTs) were consistent throughout operation. Reactors had average RTs of 2.8, 4.3, 5.7, and 9.1 days for *M. album* BG8 reactors and 2.8, 4.3, 5.8, and 9.0 days for *M. parvus* OBBP (see Fig. S1 in the supplemental material). RTs ensured that the methanotroph cultures in these reactors could reach distinct steady-state growth conditions. Reactors were considered steady state when biomass, oxygen, and methane levels were not changing significantly. All reactors reached steady-state conditions at all four RTs (Fig. 1); duration, average biomass and substrate levels, and methane oxidation rates for steady-state periods are provided in Tables S1 and S2.

Steady-state periods of *M. album* BG8 reactors are provided in Table S1 and shown in Fig. 1A and B. Dissolved-methane concentrations ranged between 0.21 and 1.68 mg L^−1^, and oxygen concentrations ranged from 3.77 to 5.26 mg L^−1^, decreasing with increasing retention time. Steady-state periods of the *M. parvus* OBBP reactors are provided in Table S2 and shown in Fig. 1C and D. Dissolved-methane concentrations ranged between 0.42 and 2.16 mg L^−1^, while oxygen concentrations varied only slightly across all RTs, ranging from 4.19 to 5.41 mg L^−1^. Both cultures followed the trend of increasing biomass with increasing retention time. Overall, biomass levels for *M. album* BG8 reactors were higher than for *M. parvus* OBBP for reactors with equivalent retention times. Differences in biomass amounts could be from differences in growth rate, internal carbon storage, and intracytoplasmic membrane (ICM) amounts, which differ between type I and type II methanotrophs (38). Methane levels observed during substrate limitation periods for both cultures were not always below reported methanotroph half-saturation constant (*K_S_*) values reported in the literature, which range from 0.06 to 1.48 mg L^−1^ (39–41). However, the observed levels were enough to affect methanotroph growth and activity. It is known that *K_S_* values are system specific (e.g., impacted significantly with stirring rate) and could be higher in the case of the methanotrophs grown in the membrane bioreactors. Volume-normalized methane oxidation rates were consistent across retention times and cultures (0.095 to 0.105 mg CH_4_ mL^−1 ^day^−1^) (Tables S1 and S2). Biomass-normalized methane oxidation rates decreased with increasing retention times for both cultures, reflecting the increase in biomass with retention time. Reactors had similar overall methane consumption rates while having distinct biomass levels. Differences in biomass-normalized methane oxidation rates implied different activities of the reactor methanotroph populations. Methane oxidation rates in this study agreed with reported literature values of 0.00014 to 0.14 mg CH_4_ mL^−1 ^day^−1^ (42) and 0.48 to 3.85 mg CH_4_ mg cell (dry weight)^−1 ^day^−1^ (37, 43) for aerobic methanotrophs.

### Biomarker amounts and oxidation rates under steady-state conditions.

**FIG 2:**
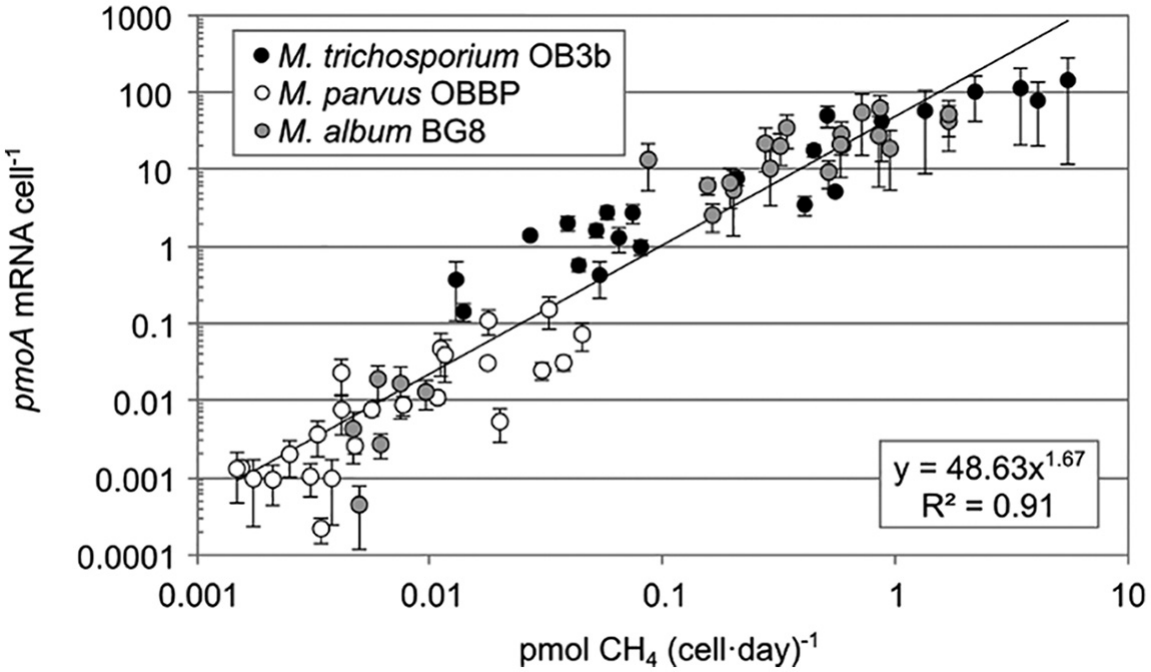
Steady-state per-cell *pmoA* transcript amounts and methane oxidation rates for aerobic methanotrophs. Data are averages from individual reactors from distinct sampling dates. Error bars represent standard deviations of biomarker amounts (*y* axis) from replicate reactors. Power law trend and *R*^2^ value are shown. *M. trichosporium* OB3b data were obtained from the work of Tentori and Richardson (37).

Correlations between steady-state methane oxidation activity and cell and *pmoA* transcript amounts for *M. album* BG8 and *M. parvus* OBBP reactors were determined for the sample times indicated in Tables 1 and 2 and Fig. S2. For both methanotrophs, reactor methane oxidation rates and *pmoA* transcript amounts were normalized by corresponding genome amounts to obtain per-cell methane oxidation rates and per-cell *pmoA* transcript levels (Fig. 2).

**TABLE 1:** Detailed operating conditions for *M. album* BG8 reactors

Retention time (days)	Condition	Time (days)	Operational change	Times when samples were collected
2.8 ± 0.1	Steady state	0–15.2		Days: 12, 13,^*a*^ and 15
	CH_4_ limitation	15.2–16.2	CH_4_ off	Hours after CH_4_ off: 0.5, 1, 2,^*a*^ 8, 12, and 24
	Recovery	16.2–17.2	CH_4_ on	Hours after CH_4_ on: 8 and 24
5.7 ± 0.1	Steady state	17.2–38.8	RT increase	Day: 30
4.3 ± 0.1	Steady state	0–15.2		Days: 12, 13,^*a*^ and 15
	O_2_ limitation	15.2–16.2	O_2_ off	Hours after O_2_ off: 0.5, 1, 2,^*a*^ 8, 12, and 24
	Recovery	16.2–17.2	O_2_ on	Hours after O_2_ on: 8 and 24
9.1 ± 0.2	Steady state	17.2–38.8	RT increase	Day: 30

a Samples were selected for RNA-seq.

**TABLE 2:** Detailed operating conditions for *M. parvus* OBBP reactors

Retention time (days)	Condition	Time (days)	Operational changes	Days when samples were collected
2.8 ± 0.1	Steady state	0–19.5		18, 19
5.8 ± 0.2	Steady state	19.5–36.2	RT increase	32, 34
4.3 ± 0.2	Steady state	0–19.5		17, 18, 19
9.1 ± 0.4	Steady state	19.5–44.0	RT increase	36, 43

The range of per-cell *pmoA* transcript levels across oxidation rates suggests a wide range of activities for the methanotroph cultures in this study. *M. album* BG8 cultures exhibited a wider range of both per-cell transcript amounts (0.0004 to 60.84) and per-cell methane oxidation rates (0.005 to 1.681) than did *M. parvus* OBBP, 0.0002 to 0.150 and 0.003 to 0.045, respectively. The observed per-cell *pmoA* transcript levels for these cultures are similar to those in *M. trichosporium* OB3b cultures (0.25 to 120.74) grown in the same type of reactor (37). They are also comparable to other reported per-cell *pmoA* transcript amounts under controlled (21, 44–46) and *in situ* (35, 36, 47) conditions and reported per-cell methane oxidation rates (17, 22, 48, 49). Higher per-cell *pmoA* transcript levels matched well with cultures with shorter RTs for both *M. album* BG8 and *M. parvus* OBBP. Increased per-cell *pmoA* transcript amounts are hypothesized to correspond to more active cells with increased activities. For the three aerobic methanotroph cultures in Fig. 2, a strong positive correlation (Pearson’s *R*^2^ = 0.91) between per-cell *pmoA* transcript levels and per-cell methane oxidation rates was observed across several orders of magnitude. Strong correlations were maintained when pure methanotroph cultures were examined individually (Fig. S3). Cell amounts alone and methanotrophic activity have been shown to be poorly correlated previously in methanotrophs (37, 50). Identical populations in terms of cell abundances can display vastly different activities, which could explain the stronger relationship when looking at cell amounts in conjunction with transcript amounts. The strong correlation observed for per-cell *pmoA* transcript levels and per-cell methane oxidation rate is due to the regulation of pMMO in response to available methane for oxidation. These results, spanning three aerobic methanotroph species, including both type I and type II methanotrophs, demonstrate that per-cell *pmoA* transcript levels may serve as a universal quantitative biomarker of extant bacterial methanotrophic activity. To date, this correlation has only been demonstrated under controlled lab conditions using pure cultures and requires more robust testing in complex microbial bioreactors and ecosystems.

### *M. album* BG8 reactor response to substrate limitation.

**FIG 3:**
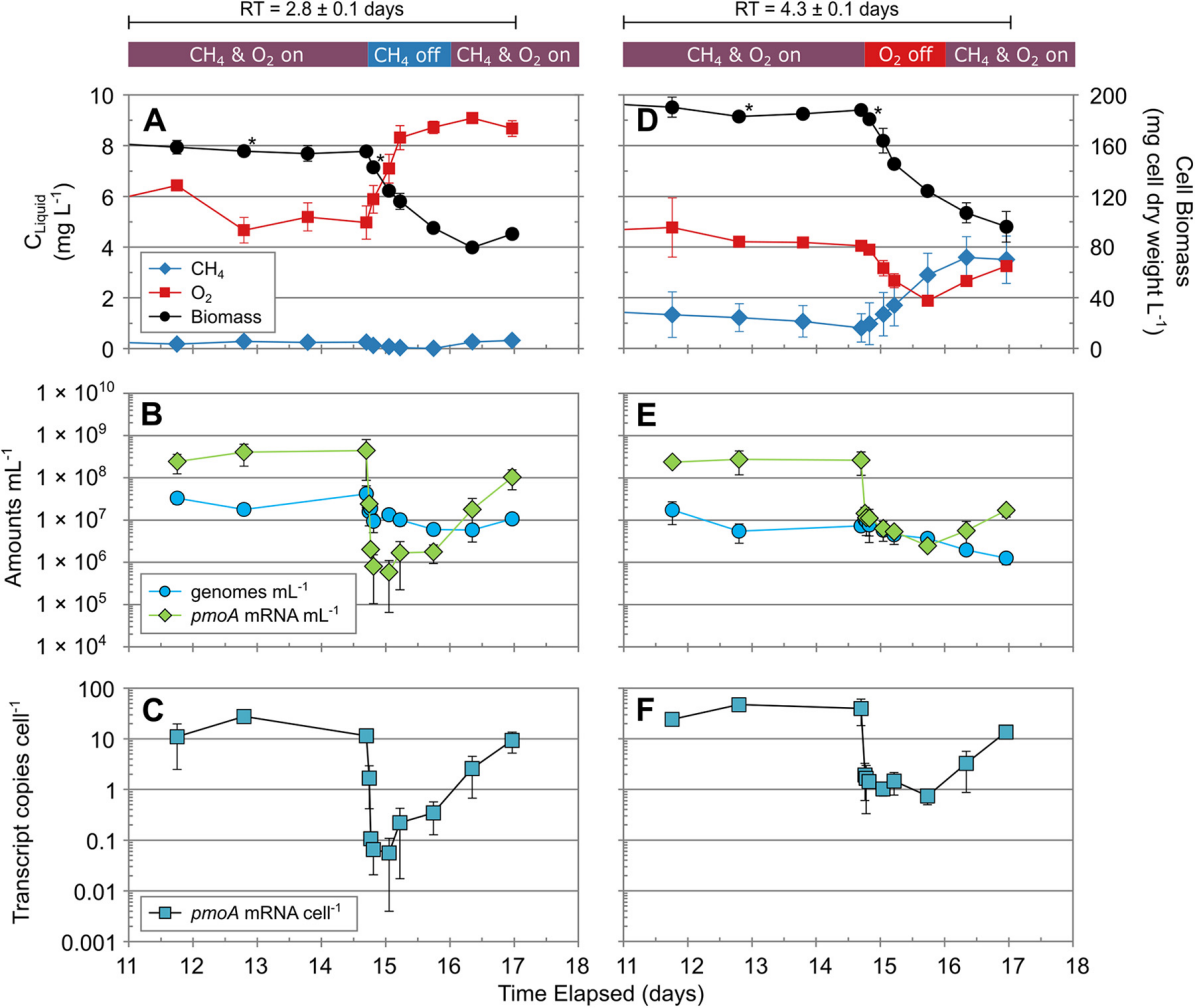
*M. album* BG8 reactors during substrate limitation and recovery. (A) 2.8-day RT reactor response during CH_4_ limitation; (B) 2.8-day RT reactor genome and *pmoA* mRNA amounts during CH_4_ limitation; (C) 2.8-day RT reactor *pmoA* mRNA copies per cell during CH_4_ limitation; (D) 4.3-day RT reactor response during O_2_ limitation; (E) 4.3-day RT reactor genome and *pmoA* mRNA amounts during O_2_ limitation; (F) 4.3-day RT reactor *pmoA* mRNA copies per cell during O_2_ limitation. Data are means ± standard deviations from triplicate reactors. Asterisks indicate samples selected for RNA-seq. Data following RT switch are not shown.

At 15.2 days of operation, effects of a 24-h methane and oxygen limitation on *M. album* BG8 cultures were explored. The limitation period was followed by a 24-h recovery period in which membrane pressures were turned back on (Fig. 3 and Fig. S2A and B). Methane limitation caused reactor oxygen levels to increase from 5.5 to 9.0 mg L^−1^ due to the lack of incoming methane, while biomass levels decreased by about half (Fig. 3A). The decrease in biomass was reflected in both cell genome and *pmoA* mRNA amounts (Fig. 3B). Within 2 h of methane limitation, genome copies were about a third of steady-state amounts, while *pmoA* mRNA amounts decreased by about 3 orders of magnitude. During steady state, per-cell *pmoA* transcript levels ranged from 9.02 to 11.60 transcripts per cell. A sharp decrease in the *pmoA* transcriptional activity 0.5 h after the onset of methane limitation was observed, reaching its minimum 8 h postlimitation, with a log_2_ fold decrease of 8.2 in per-cell *pmoA* transcript levels compared to average steady-state levels (Fig. 3C and Table S3). Per-cell *pmoA* transcript levels stayed significantly depleted through the 24 h following the onset of methane limitation. Resumption of methane saw sharp increases in *pmoA* transcript amounts, with per-cell *pmoA* transcript levels approaching steady-state levels 24 h after recovery, while biomass (as both genome copies and dry cell weight) lagged (Fig. 3B and C and Table S3).

In oxygen-limited reactors, a sharp decrease in biomass levels was also observed, which continued into the recovery period (Fig. 3D). Steady-state *pmoA* transcript levels for the 4.3-day reactors ranged between 24.19 and 47.52 for the days preceding oxygen limitation. During oxygen limitation, oxygen levels decreased from 4 to 2 mg L^−1^ and methane levels increased from 1 to 3 mg L^−1^. Genome copies dropped slowly throughout both the limitation and recovery periods, while *pmoA* mRNA amounts decreased by 2 orders of magnitude 0.5 h after oxygen limitation, with a more gradual decline observed in the subsequent hours during oxygen limitation (Fig. 3E). The decrease pattern in *pmoA* transcript amounts was reflected in per-cell *pmoA* transcript levels, with a log_2_ fold decrease of 4.3 in compared to average steady-state levels (Fig. 3F and Table S3). The oxygen recovery period also saw increases in *pmoA* transcript amounts which led to per-cell *pmoA* transcript levels comparable to steady-state levels after 24 h following oxygen repletion (Fig. 3B and C and Table S3).

The *M. album* BG8 biomarker response observed due to methane and oxygen limitation differed compared to that in steady state. Genome copies in the methane-limited reactors exhibited a sharp decrease within the first hour, with no further change observed through 24 h until recovery, when a small increase in genome copies was observed, while oxygen-limited reactors exhibited a steady decrease throughout both the limitation and recovery periods (Fig. 3B and E). Both methane and oxygen limitation had the largest decrease of *pmoA* transcript levels in the first hour, with a larger overall decrease observed with methane limited. Differences between per-cell *pmoA* transcript levels during both methane and oxygen limitation and their preceding steady-state levels were statistically significant (*P < *0.001) (Table S4). Both sets of reactors reached per-cell transcript levels comparable to steady-state averages within the 24-h recovery period. Differences in per-cell *pmoA* transcript levels were statistically significant during methane limitation and recovery (*P < *0.01) and during oxygen recovery and the preceding limitation period (*P < *0.05) (Table S4). The impact of oxygen limitation may have been less than that of methane limitation, as oxygen was present in the reactor influent. Similar *pmoA* expression patterns have been observed in mixed-consortium bioreactors, where cultures grown under feast conditions showed 2- to 3-fold per-cell *pmoA* transcript decreases within 1 h of famine conditions (51). Rodríguez et al. (51) also observed that per-cell *pmoA* transcripts of methanotroph cultures regularly exposed to recurring feast-famine conditions were not affected by the onset of famine, seeing no change in the first 12 h. Overall *pmoA* mRNA per-cell levels observed in the current study were orders of magnitude higher than in the study by Rodríguez et al. (0.00005 to 0.002) (51) and in line with those of *M. trichosporium* OB3b cultures (37, 52) and Methyloprofundus sedimenti (21). The observed decrease in per-cell *pmoA* transcripts during the first hours of methane limitation suggest a fast *pmoA* regulation in response to methane levels. This is also supported by the increase observed in per-cell *pmoA* transcripts when methane was available during the recovery period, where a response occurred in the first 8 h and reached steady-state levels after 24 h (Fig. 3C). The observed rapid response of methanotrophs to changing methane and oxygen conditions suggests that they can quickly adapt to changes in local environmental conditions.

In addition to cell amounts decreasing, the observed changes in biomass could be due to decreases in protein mass, as starvation under aerobic conditions has been demonstrated to induce protein biomass loss in methanotrophs (17). Rapid changes at the transcript level due to changes in local conditions are well documented for aerobic methanotrophs. The “copper switch,” a well-established link between copper levels and expression of pMMO or sMMO, has been shown to occur in the order of minutes to hours (23, 53, 54). Aerobic methanotrophs have also been shown to regulate expression of methane oxidation pathways in response to the type and amount of carbon source available (e.g., methane versus other one-carbon compounds) (55–57). This suggests that decreased methane amounts during periods of limitation would lead to a decrease in *pmoA* expression and a decrease in *pmoA* transcripts per cell and the opposite effect during the recovery period. Rapid (<2-h) responses from methanotrophs to starvation and recovery periods when grown in different biofilters and stirred tank reactors have been observed (51, 58). Rodríguez et al. grew methanotroph reactors under different operational methods, feast-famine growth, and continuous growth and examined the influence of operational period on performance (methane consumption) and *pmoA* expression levels. Following a famine period, reactors under both operational methods were able to recover to their prelimitation performance within 2 h; however, the *pmoA* expression levels were influenced by operational mode, with a more drastic decrease observed in *pmoA* expression levels for the continuous-growth reactors (51). However, different trends have been observed in the methanotroph *M. sedimenti*, for which short-term methane starvation periods led to increased *pmoA* expression throughout the starvation period, with the opposite trend observed during recovery (21). Differing expression patterns between methane oxidation and the methanol dehydrogenase (MDH) genes *mxaF* and *xoxF* have been observed during starvation, suggesting independent regulation (21). The differences in expression observed between methanotrophs could serve as different survival strategies during starvation periods.

### *M. album* BG8 transcriptomic samples and gene expression response to short-term substrate limitation.

Transcriptome sequencing (RNA-seq) samples from *M. album* BG8 under short-term substrate limitation yielded 170.4 million reads across the 8 multiplexed samples from the four conditions (Table S5). Gene counts showed agreement after normalization, with medians consistent across samples and between biological replicates (Fig. S4). Biological replicates were examined using principal-component analysis (PCA) of normalized logarithmic transformed read counts using DESeq2 (59). Similarity was observed between duplicate biological replicates for the substrate-limiting conditions. Steady-state samples showed less uniformity due to one replicate from the 4.2-day RT (Fig. S5). Despite the variability observed in PCA of the *M. album* BG8 transcriptome samples under steady state, samples were not considered outliers, as no significant differences in transcripts were detected. Coverage and normalized counts were similar across replicates, and replicates for all conditions were included in subsequent analyses.

**FIG 4:**
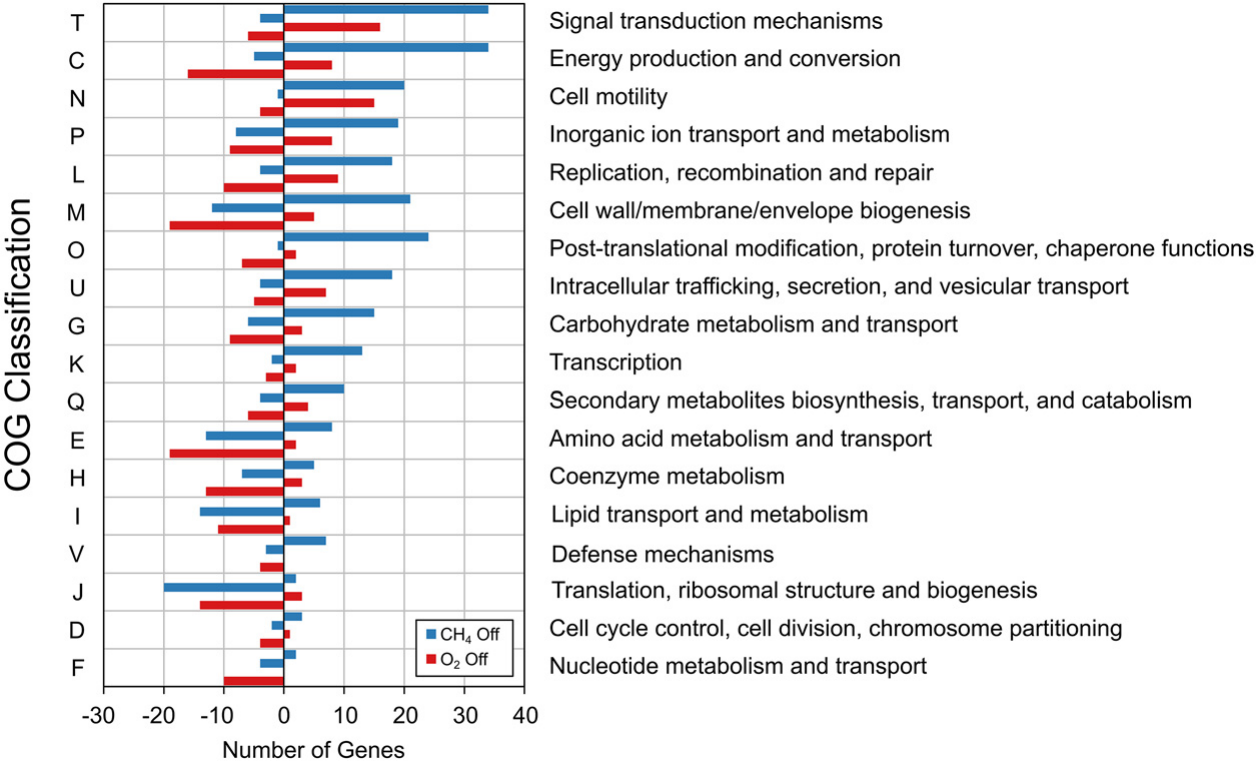
Classification of significant differential gene expression (DGE) in *M. album* BG8, based on COG classification from the eggNOG 5.0 database. Positive and negative values on the *x* axis indicate numbers of genes in that COG category that were upregulated and downregulated, respectively, compared to steady-state conditions. Unclassified, category S (“Function Unknown”) and categories with fewer than two genes with DGE are not shown.

Collectively across all transcriptomes, expression was observed for 3,931 out of 3,984 (98.7%) of predicted protein-coding genes in the published *M. album* BG8 genome (60). Significant differential gene expression (DGE) (log_2_ fold change [FC] > | 1.0 |; adjusted *P* value [*P*_adj_] < 0.05) was observed for CH_4_- and O_2_-limited samples compared to corresponding steady-state samples (Table S6 and Data Set S2). Methane limitation and oxygen limitation resulted in 444 and 282 genes, respectively, with significant DGE compared to reference steady-state samples. Reference steady-state samples had similar expression profiles regardless of 2.8- or 4.2-day RT, with only 4 genes showing significant DGE. EggNOG 5.0 was used to categorize genes showing DGE between substrate-limited and corresponding steady-state samples, categorizing 74% and 81% of differentially expressed genes of the methane- and oxygen-limited samples, respectively (Fig. 4).

Both CH_4_- and O_2_-limited conditions triggered upregulation of several of genes related to cell motility, flagella, and chemotaxis (identifier in Clusters of Orthologous Groups [COG] database: N) (Fig. 4 and Data Set S2). Upregulation of motility genes has been linked to nutrient limitation, with motile cells seeking more favorable conditions (61). In methanotrophs, growth conditions influence expression of motility and adhesion genes. Similar expression patterns have been observed in *M. album* BG8 and *Methylocystis* sp. strain Rockwell grown with methanol (62) in which flagellum genes were upregulated. In this study, both limitation of CH_4_ and limitation of O_2_ resulted in a strong consistent downregulation of translation, ribosomal structure, and biogenesis (COG: J) genes, with effects on ribosomal protein genes more pronounced in methane limitation, while oxygen limitation mostly affected translational genes (Fig. 4 and Data Set S2).

The changes in expression observed, with an increase in cell motility, posttranslational modifications, and signal transduction genes and a decrease in carbohydrate, amino acid, and lipid metabolism and transport, and translation genes, are similar to those observed for *M. album* BG8 grown with methanol compared to methane (62).

For all conditions, genes involved in methane oxidation and methanol oxidation were among the most highly expressed genes (16.6 to 28.9% of all transcripts [Data Set S2]), following expression trends observed in aerobic methanotrophs (63–65). The general trend was downregulation in C_1_ metabolism genes upon short-term limitation. Exceptions were upregulation of alcohol dehydrogenase gene *xoxF* under both conditions and formate dehydrogenase (*fdh*) specifically during methane limitation. However, the C_1_ metabolism gene transcript remained among the most highly abundant reads detected even during stress. A period of substrate limitation longer than 2 h might be required to observe an effect due to elevated starting transcript amounts for these pathways and the half-life of RNA. Partially degraded RNAs may still being “readable” by the RNA-seq method, unlike with qPCR, in which full-length transcripts are required for detection.

**FIG 5:**
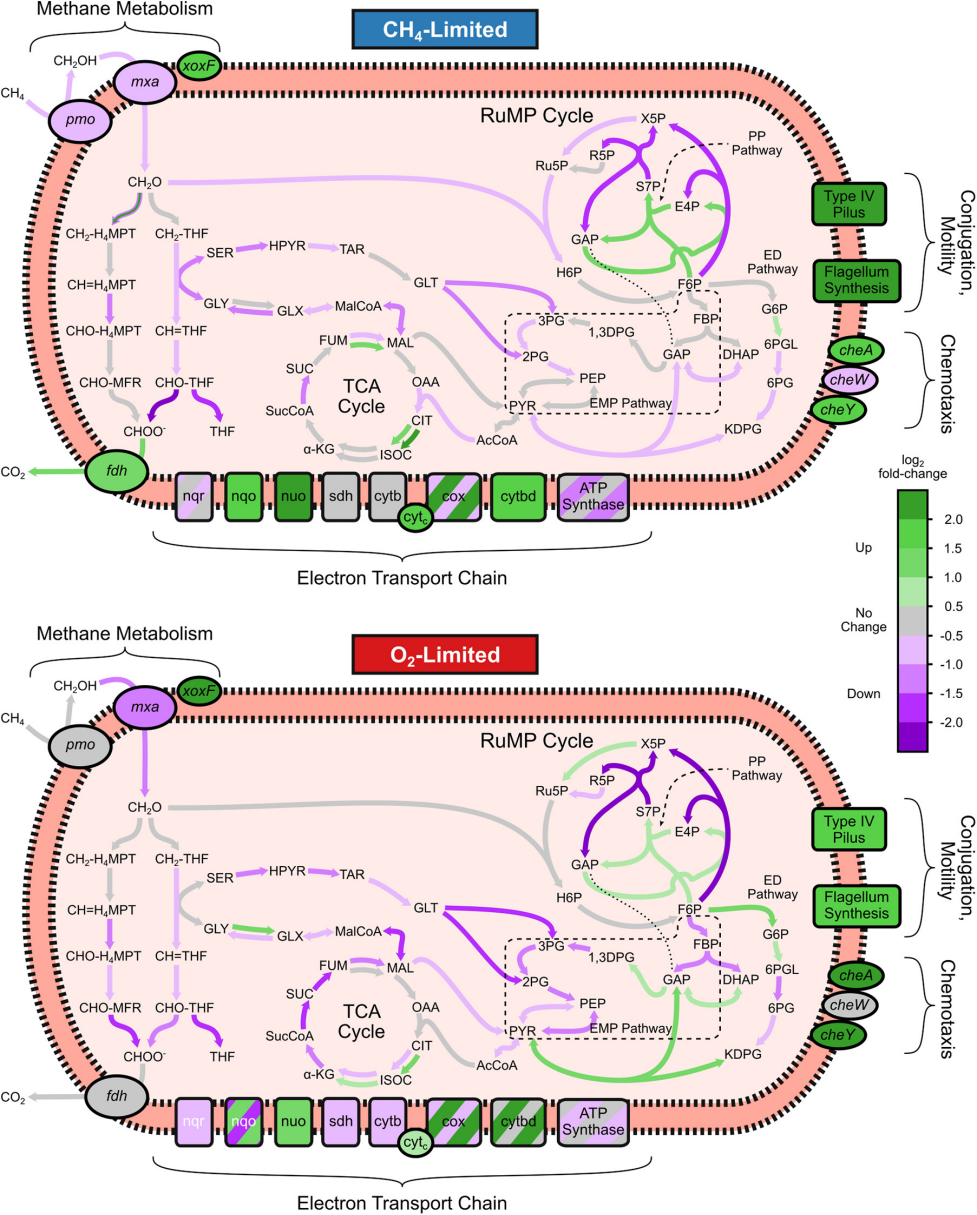
Changes in central metabolic pathways in *M. album* BG8 during short-term substrate limitation of CH_4_ (top) and O_2_ (bottom). Upregulated (green) and downregulated (purple) pathways and pathways with no change (gray) compared to steady-state conditions are highlighted. Multiple colors indicate that different genes in that pathway were both up- and downregulated. Abbreviations and corresponding intermediates and compounds are provided in Table S7.

A strong effect was observed in genes involved in energy production and conversion (COG: C) (Fig. 4). For methanotrophs, these changes in expression of genes for energy production and conversion are hypothesized to be in response to the available substrate, to compensate for differences in energy available and minimize effect on core metabolic pathways (66). A visual representation of the response of central metabolic pathway genes and other genes of interest to methane and oxygen limitation is shown in Fig. 5. NADH oxidoreductase and cytochrome genes were upregulated under methane-limited conditions, while ATP synthases were downregulated, with stronger effects observed under CH_4_ limitation. Upregulation of cytochrome oxidase genes in response to O_2_ limitation was observed, and such upregulation has been previously observed in Methylomicrobium buryatense 5GB1C (20); however, a stronger response was observed for CH_4_ limitation. Different carbon sources have been found to affect transcriptional responses of both alpha- and gammaproteobacterial methanotrophs (55, 67).

**FIG 6:**
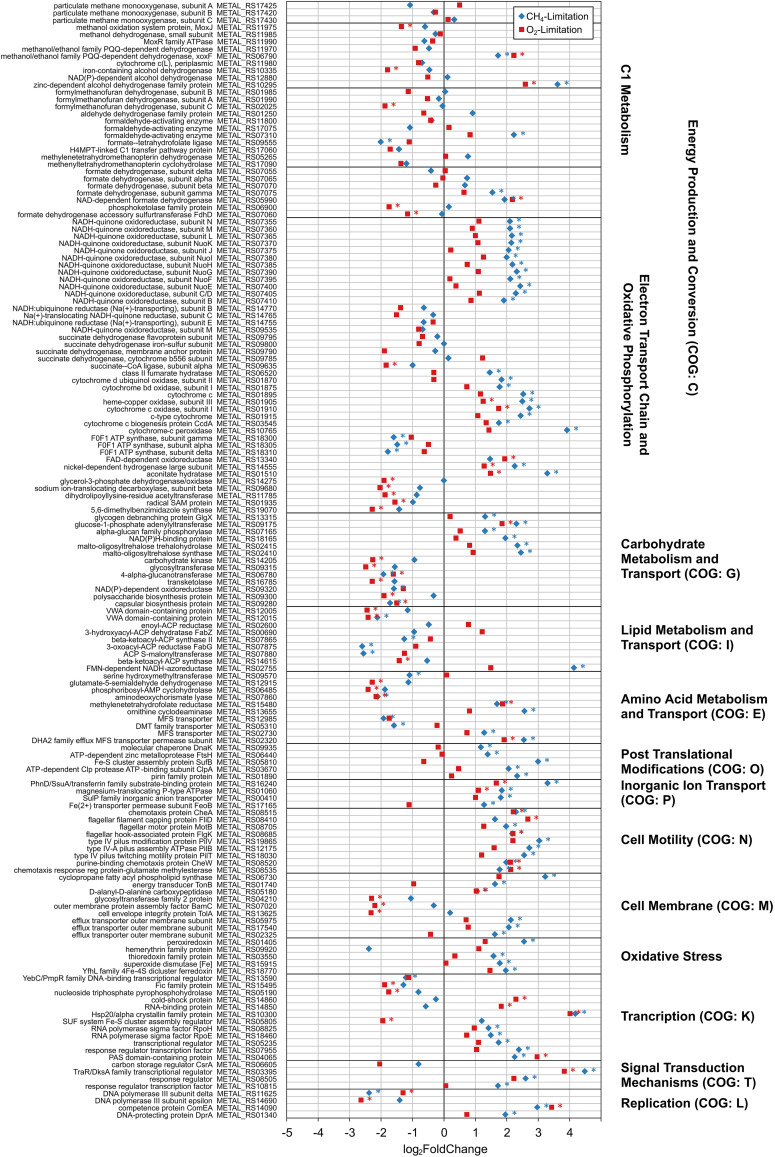
Gene expression profiles of *M. album* BG8 under short-term substrate (O_2_ and CH_4_) limitation compared to steady-state conditions. Statistically significantly differentially expressed genes (log_2_ FC > | 1.0 |, *P*_adj_ < 0.05) are indicated by asterisks. Genes are grouped by orthology and/or function. Subset of genes shown; see Data Set S2 for complete differential expression analyses.

*M. album* BG8 upregulated pentose phosphate (PP) pathway genes and electron transport chain (ETC) genes, namely, NADH oxidoreductase (*nuo*) and cytochrome genes, to adapt methane limitation while downregulating other metabolic activity (Fig. 5 and 6). During oxygen limitation, Entner-Doudoroff (ED) pathway genes, in addition to PP pathway genes, were upregulated, while Embden-Meyerhof-Parnas (EMP; glycolysis) pathway genes were downregulated and electron transport chain genes saw both slight up- and downregulation. Genes involved in oxidation of methane (*pmo*) and methanol (*mxa*) were generally downregulated under both conditions. A homolog of methanol dehydrogenase, XoxF, was highly upregulated under both limiting conditions. XoxF-type methanol dehydrogenases, however, are lanthanide dependent (68), and reactor medium was unchanged, indicating that this could be a survival response upon nutrient limitation or to shift metabolism entirely during stress conditions. These could be potential strategies for maintaining activity and growth upon substrate limitation. Research on *M. buryatense* 5GB1 and *M. album* BG8 grown on methanol suggests that electrons obtained from methanol dehydrogenase are directly transferred to cytochromes and the electron transport chain, bypassing the electron requirements from NADH oxidoreductase complex for ATP production (67). Some studies have shown that type I methanotrophs potentially perform fermentation-based methanotrophy, in which methane-derived formaldehyde can be used for formation of formate, acetate, succinate, lactate, and hydroxybutyrate under low-oxygen conditions (69). In Methylomicrobium alcaliphilum 20Z, low oxygen induced increased expression of EMP genes, predicted fermentation pathway enzyme genes and O_2_ carrier hemerythrin, and decreased expression of NADH:ubiquinone oxidoreductase and cytochrome *c* oxidase in addition to detection of possible fermentation products and H_2_ (69). The genome of *M. buryatense* 5GB1 also contains homologs of enzymes required for fermentation, and its metabolism under O_2_ limitation is a combination of fermentation and respiration (20). Another potential strategy during substrate limitation is use of internal carbon storage. *M. album* BG8 has genes for glycogen biosynthesis, just as does “*Candidatus* Methylacidiphilum fumariolicum” SolV from phylum *Verrucomicrobia*, which consumes stored glycogen during periods of substrate limitation (19). In this study, periods of both methane and oxygen limitation led to significant downregulation of glycosyltransferase and glycogen synthase genes and an upregulation in glycogen debranching and cleaving genes (COG: G), which are potentially involved in glycogen synthesis and degradation, respectively (Fig. 6).

Short-term substrate limitations led to transcriptional changes in ETC enzyme genes, preference for the PP pathway, and, in the case of O_2_ limitation, upregulation of ED pathway genes. These responses show potential strategies where pathways are shifted to use internal carbon reserves and, potentially, fermentation for survival. In addition to the changes in carbon metabolism, observed changes in gene expression in both methane- and oxygen-limited samples were indicative of methanotrophs with decreased activity (70), and short-term substrate limitation was sufficient to elicit a stress response. DGE genes and additional genes of interest grouped by COG for substrate-limited samples are shown in Fig. 6.

Additionally, both limitation conditions had downregulation of the carbon storage regulator, *csrA*, and significant upregulation of sigma factor *rpoE*, an expression pattern indicative of stress response (71). DGE was observed for other genes indicative of stress response as seen for stressed *M. album* BG8 grown with methanol (67) (Fig. 6). Methane limitation led to upregulation of oxidative stress genes, including superoxide dismutase (log_2_ FC = 1.8; *P*_adj_ < 0.05), peroxiredoxin (log_2_ FC = 2.5; *P*_adj_ < 0.05), and thioredoxin (log_2_ FC = 1.6; *P*_adj_ < 0.05) genes, likely to deal with increased oxidative stress as dissolved-oxygen levels increased after methane limitation (Fig. 6). Under O_2_-limited conditions, a 2-fold increase was observed for hemerythrin, a putative O_2_-scavenging protein (72) which is upregulated in Methylomicrobium buryatense 5GB1C (20) and Methylomicrobium alcaliphilum 20Z (69) under conditions of oxygen limitation. Additional stress indicators were downregulation of DNA polymerase genes and upregulation of competence and DNA-protecting protein genes (COG: L) (Fig. 6). In addition to transcriptional regulation, shifts in core metabolic processes could be regulated via translational and posttranslational mechanisms as well (66).

The 2-h substrate limitation period was enough to observe a transcriptional response in *M. album* BG8 under both conditions. Experimental and modeling results have shown that transcriptional responses in bacteria due to substrate limitation are fast, being elicited within 1 h, while translational changes may continue for more than 10 h (73). When compared to steady-state levels, RNA-seq data showed *pmoA* transcript amounts decreased about 2-fold during substrate-limiting conditions. However, the effects were not as drastic as observed in reverse transcription-qPCR (~100-fold decrease). The observed difference could be due to partially decayed transcripts being read in RNA-seq, whereas for reverse transcription-qPCR, only the nondegraded full *pmoA* target region would be amplified and detected.

This work highlights mRNA biomarkers as indicators of methanotrophic activity as well the transcriptomic response of *M. album* BG8 to short-term substrate limitations, demonstrating a fast response to dynamic conditions and potential strategies for growth under periods of limited substrate availability. The results presented suggest a cross-genus, potentially universal trend between steady-state per-cell activity and per-cell *pmoA* transcript levels in aerobic bacterial methanotrophs across several orders of magnitude. Universal trends would only be possible if these correlations are explored in anaerobic, bacterial methanotrophs from phylum NC10 and *Verrucomicrobia*. This work holds promise for assessing methanotroph growth and activity in diverse environments and informs operational strategies in biotechnological applications of methanotrophs, for example, in biorefinery scenarios where biogas is upcycled into more valuable bioproducts.

## MATERIALS AND METHODS

### Bacterial strains, reactor design, and experimental conditions.

Experiments were conducted at 25°C using pure cultures of *M. parvus* strain OBBP (NCIMB 11129) and *M. album* strain BG8 (NCIMB 11123) grown on nitrate mineral salts (NMS) medium (74). Prior to inoculation of reactors, methanotroph cultures were grown in crimp-capped serum bottles as previously described (37). Bioreactors consisted of modified 1-L Pyrex medium bottles (Corning, Corning, NY), with a 0.80-L liquid volume and 0.27-L headspace volume and with two sets of silicone tubing serving as membranes for bubbleless delivery of CH_4_ (≥99.5% purity; Airgas, Radnor, PA) and O_2_ (≥99.99% purity; Airgas), as previously described (37). Reactors were operated as chemostats, continuously provided NMS medium, and RTs were determined by measuring reactor effluent, as previously described (37). During periods of substrate limitation for *M. album* BG8 reactors, membrane pressures were disconnected from gas tanks and opened to room atmosphere. Two sets of triplicate reactors of *M. album* BG8 (Table 1) and *M. parvus* OBBP (Table 2) cultures each were started concurrently with initial RTs of 2.8 and 4.3 days.

Reactors were operated under the initial conditions until methane and biomass levels stabilized, considered steady-state operation. This initial periods were approximately 15 days (3 to 5 retention times) for *M. album* BG8 reactors and 19.5 days (>4 RTs) for *M. parvus* OBBP reactors. Following the steady-state period, the methane and oxygen supplies were shut off in the *M. album* BG8 2.8-day and 4.3-day reactors, respectively, for a 24-h period of substrate limitation. After 24 h, methane and oxygen were turned back on for a 24-h recovery period, before the *M. album* BG8 2.8- and 4.3-day RT reactors were transitioned to longer retention times of 5.8 and 9.1 days, respectively. *M. album* BG8 reactors were operated to reach a new steady state (2.5 to 3 RTs). After 19.5 days (>3 RTs), *M. parvus* OBBP reactors were transitioned to retention times of 5.8 and 9.1 days and operated until steady-state operation was achieved. Diagrams showing reactor operation and sampling times of both cultures are provided in Fig. S2.

### Methane, oxygen, biomass sampling, and methane oxidation rate measurements.

Reactor methane, oxygen, and microbial biomass concentrations were monitored throughout operation. Methane and oxygen concentrations were determined from reactor headspace measurements using gas chromatography with a thermal conductivity detector (GC-TCD) and Henry’s law coefficient, and microbial biomass was monitored via readings for optical density at 600 nm (OD_600_) and converted to cell biomass (dry weight) as previously described (37). Biomass samples for nucleic acid extraction were collected on selected dates during both steady-state and substrate limitation periods (Tables 1 and 2). Methane oxidation rates were determined using reactor methane and biomass data, available organism kinetic parameters (12, 40, 75), and a mechanistic model describing the membrane bioreactor system (37). Reactor methane oxidation rates (milligrams of CH_4_ per liter per day) were converted to obtain methane oxidation rates per unit volume, and cell normalized methane oxidation rates were obtained using determined *pmoA* gene copies and *pmoA* copies per genome for each organism (1 copy for *M. album* BG8 [60] and 2 copies for *M. parvus* OBBP [76]).

### Nucleic acid extraction, cDNA synthesis, and qPCR analyses.

Sample collection, extraction, storage, and processing were done as previously described (37). Nucleic acid quality assurance/quality control (QA/QC) was performed at the Cornell Biotechnology Resource Center (BRC). Populations (genome copies) and activities (*pmoA* transcripts) of *M. parvus* OBBP and *M. album* BG8 cultures were monitored via qPCR and reverse transcription-qPCR, respectively, targeting gene *pmoA* (subunit A of pMMO) using previously published general aerobic methanotroph primer set A189F/Mb661R (24, 77). Luciferase (*luc*) DNA and mRNA (Promega, Madison, WI) spike-ins were used as internal standards to correct for losses during extraction and reverse transcription (78), using previously described methods (37). All qPCRs were performed in triplicate 20-μL reaction mixtures using Luna universal qPCR master mix (New England Biolabs) on an iCycler IQ multicolor real-time detection system (Bio-Rad), with contamination checks and verification of correct qPCR product performed as previously described (24, 37, 78). Methanotroph cell amounts (as genome copies) were determined via qPCR from *pmoA* gene amounts and *pmoA* copies per genome for each organism as described above. Biomarker *pmoA* transcript levels were determined via reverse transcription-qPCR on cDNA and normalized by genome copies to obtain per-cell *pmoA* transcript amounts. Differences between *pmoA* transcript amounts per cell for steady state, substrate limitation, and recovery conditions were evaluated with the Mann-Whitney U test using R studio software (v3.4.2).

### RNA sequencing, assembly, and differential gene expression.

RNA-seq was performed on duplicate *M. album* BG8 samples at steady state (day 13) and 2 h after substrate limitation (Table 1 and Fig. S2). Samples were submitted to Cornell University’s Transcriptional Regulation & Expression Facility (TREx) for quality checks, library preparation, and RNA sequencing. RNA integrity was determined using a fragment analyzer (Advanced Analytical, Santa Clara, CA). Using a total RNA input of 500 to 1,000 ng, rRNA was subtracted by hybridization from total RNA samples using the NEBNext rRNA depletion kit for bacteria (New England Biolabs). TruSeq-barcoded RNA-seq libraries were generated with the NEBNext Ultra II directional RNA library prep kit (New England Biolabs) following the manufacturer’s instructions. Libraries were quantified with a Qubit 2.0 (double-stranded DNA [dsDNA] high sensitivity (HS) kit; Thermo Fisher), and size distribution was determined using a fragment analyzer (Advanced Analytical) prior to pooling. Libraries were sequenced on an Illumina HiSeq X Ten system (Illumina, Inc., San Diego, CA) with 2 × 150-nucleotide (nt) paired-end reads, generating at least 4 million reads per library. Illumina pipeline software was used for base calling, Sequenced reads were trimmed for 3′ adaptor sequence and low-quality sequence and filtered to remove reads of <50 nt with TrimGalore v0.6.0 (79), a wrapper for Cutadapt (80), and FastQC (81). Processed reads were mapped to the *M. album* BG8 genome using STAR v2.7.0e (82) using–quantMode GeneCounts to generate raw counts per gene. Raw counts were analyzed in R using SARTools (83) and DESeq2 v1.26.0 (59) to generate normalized counts and for statistical analysis of differential gene expression (DGE). Significance in differential expression was considered at a log_2_ fold change (FC) of >| 1.0 | and false-discovery rate (FDR)-adjusted *P* value of <0.05.

Genes with significant DGE during substrate limitation compared to steady-state conditions were mapped to functional identifiers in the Clusters of Orthologous Groups (COG) database using eggNOG 5.0 (84). Remaining unclassified genes, or genes classified as “Function Unknown,” were further assessed via the National Center for Biotechnology Information Basic Local Alignment Search Tool (NCBI-BLAST; https://blast.ncbi.nlm.nih.gov/Blast.cgi). *M. album* BG8 RNA-seq data were submitted to the NCBI Gene Expression Omnibus (GEO) database under accession number GSE188821.
