# Supplementary material for: RNA Biomarker Trends across Type I and Type II Aerobic Methanotrophs in Response to Methane Oxidation Rates and Transcriptome Response to Short-Term Methane and Oxygen Limitation in Methylomicrobium album BG8
Source: Microbiol Spectr. 2022 Jun 9;10(3):e00003-22. doi: 10.1128/spectrum.00003-22 (PMC9241951; doi:10.1128/spectrum.00003-22)
Supplement: Supplemental file 1 — Supplemental material. Download spectrum.00003-22-s0001.pdf, PDF file, 0.5 MB [file spectrum.00003-22-s0001.pdf]

SUPPORTING INFORMATION FOR:

RNA Biomarker Trends across Type I and Type II Aerobic Methanotrophs in Response to Methane Oxidation Rates and Transcriptome response to Short-Term Methane and Oxygen Limitation in *Methylomicrobium album* BG8

Egidio F. Tentori, Shania Fang, Ruth E. Richardson*

School of Civil and Environmental Engineering, Cornell University, Ithaca, NY 14853,

*Corresponding author: E-mail: eft35@cornell.edu; current address: Gradient, One Beacon St., 17th Floor, Boston, MA 02108

Number of Pages: 10

Number of Figures: 5

Number of Tables: 7

Table of Contents

List of Figures

Figure S1. *M. album* BG8 and *M. parvus* OBBP Reactor Retention Times (RTs). S3

Figure S2. Operating Conditions for *M. album* BG8 and *M. parvus* OBBP Reactors. S3

Figure S3. Steady-State Per Cell *pmoA* Transcript Amounts and Methane Oxidation Rates for Pure Aerobic Methanotroph Cultures. S4

Figure S4. Raw counts and normalized counts distributions for RNA sequencing samples. S4

Figure S5. Principal component analysis of *M. album* BG8 transcriptomic sequence data. S5

List of Tables

Table S1. *M. album* BG8 Reactor Data for Steady State Periods. S6

Table S2. *M. parvus* OBBP Reactor Data for Steady State Periods. S6

Table S3. *M. album* BG8 Reactors Log2Fold Change in per cell *pmoA* transcript amounts during Substrate Limitation and Recovery Periods compared to Steady State. S7

Table S4. Mann–Whitney U Test comparisons between per cell *pmoA* transcript amounts during steady state, substrate limitation and recovery conditions for *M. album* BG8 reactors. S7

Table S5. General properties of *M. album* BG8 Transcriptome Samples. S8

Table S6. Significant Differential gene expressions (DGE) for comparisons between CH_4 off, O_2 off steady state conditions in *M. album* BG8. S8

Table S7. Abbreviations of Compounds and Intermediates from Figure 6. S9

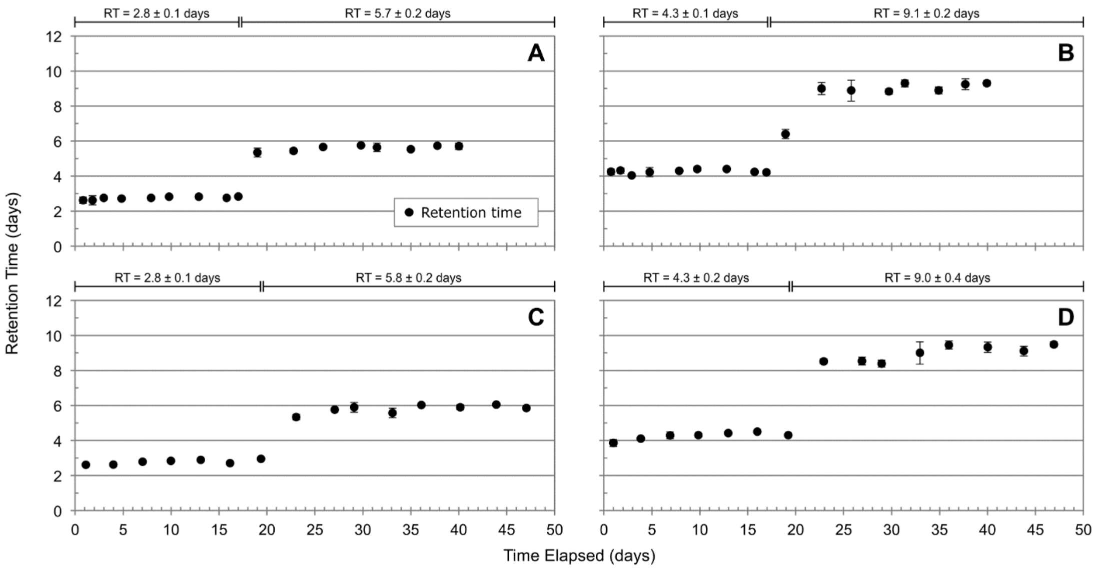

Figure S1. *M. album* BG8 and *M. parvus* OBBP Reactor Retention Times (RTs). (A) *M. album* BG8 reactors with 2.8- and 5.7-day RT. (B) *M. album* BG8 reactors with 4.3- and 9.1-day RT. (C) *M. parvus* OBBP reactors with 2.8- and 5.8-day RT. (D) *M. parvus* OBBP reactors with 4.3- and 9.0-day RT. Data are triplicate reactor averages, error bars indicate standard deviation. Top values are mean values for periods at a set RT.

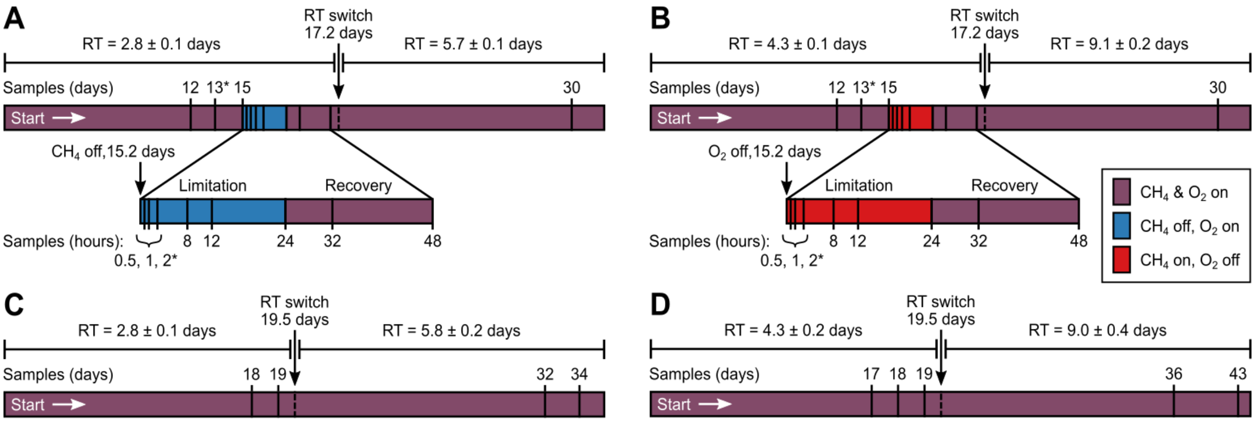

Figure S2. Operating Conditions for *M. album* BG8 and *M. parvus* OBBP Reactors. Samples for qPCR indicated by vertical lines on approximate sampling time. *M. album* BG8 RNA-seq samples indicated by asterisk (*).

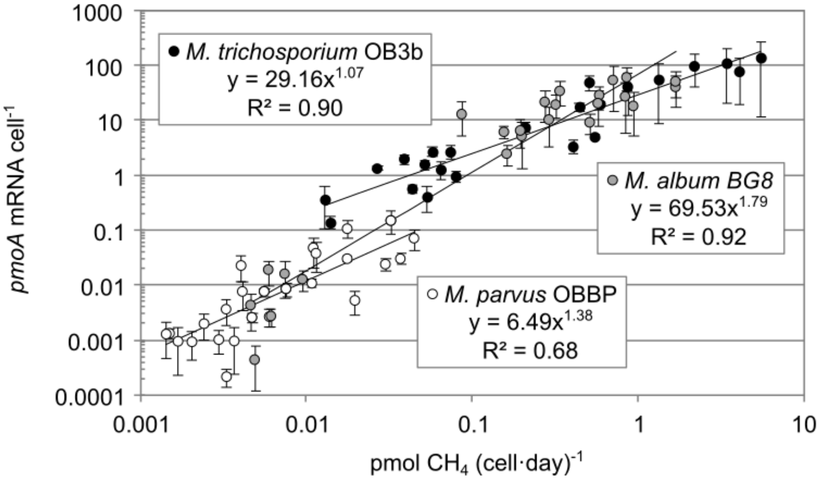

Figure S3. Steady-State Per Cell *pmoA* Transcript Amounts and Methane Oxidation Rates for Pure Aerobic Methanotroph Cultures. Data are averages from individual reactors from distinct sampling dates. Error bars represent standard deviations of biomarker amounts (y-axis) from replicate reactors. Power law trend and R^2 value are shown for each pure methanotroph culture. *M. trichosporium* OB3b data obtained from Tentori et al.(1).

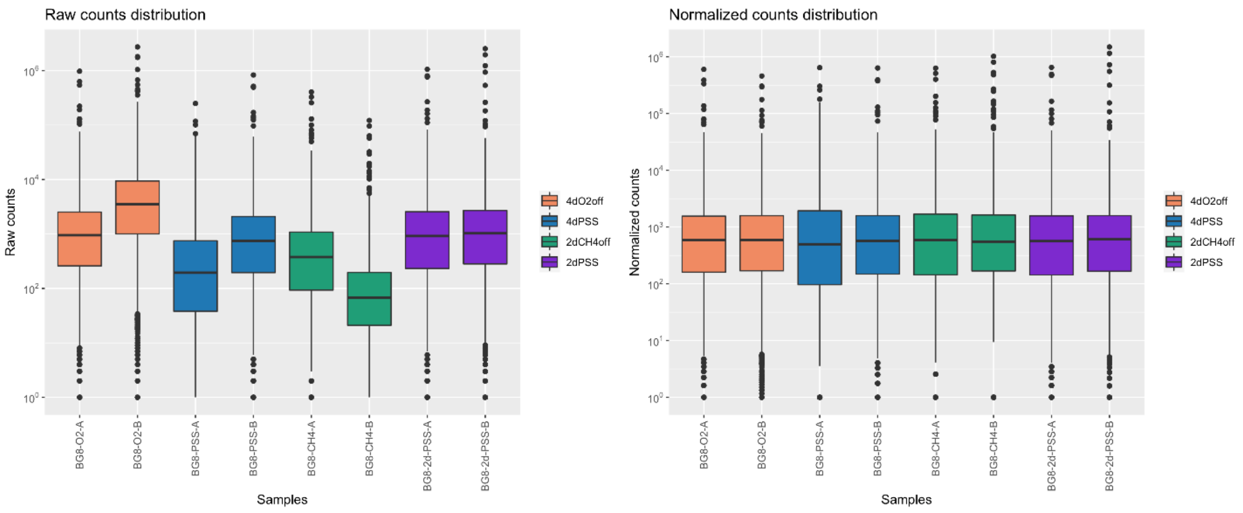

Figure S4. Raw counts and normalized counts distributions for RNA sequencing samples.

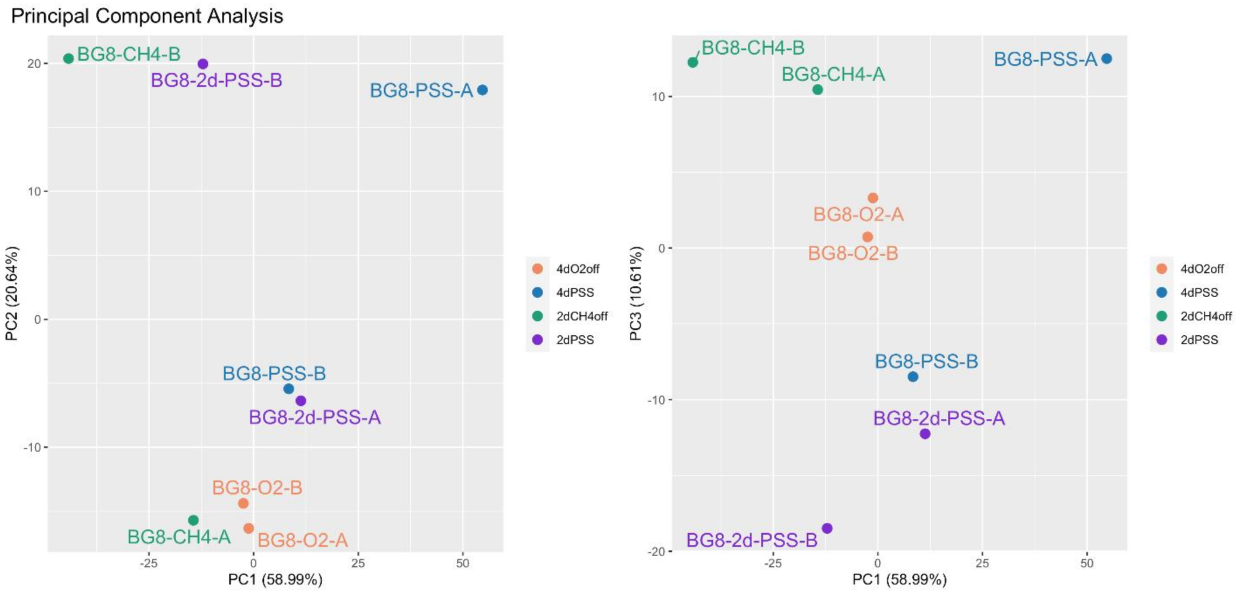

Figure S5. Principal component analysis of *M. album* BG8 transcriptomic sequence data.

Table S1. *M. album* BG8 Reactor Data for Steady State Periods.

Retention Time (days)	2.8 ± 0.1	4.3 ± 0.1	5.7 ± 0.1	9.1 ± 0.2
Steady state period (days)	6–15	8–15	24–38	25–39
CH_4 (mg L^{-1})	0.29 ± 0.09	1.36 ± 0.31	0.21 ± 0.05	1.68 ± 0.26
O_2 (mg L^{-1})	5.26 ± 0.78	4.47 ± 0.30	4.93 ± 0.39	3.77 ± 0.35
Biomass (mg cell (dry weight) L^{-1})	158.95 ± 4.41	187.10 ± 3.77	196.00 ± 8.52	231.85 ± 9.75
CH_4 oxidation rate (mg CH_4 mL^{-1} day^{-1})	0.105 ± 0.001	0.099 ± 0.002	0.105 ± 0.001	0.097 ± 0.001
CH_4 oxidation rate (mg cell (dry weight) day^{-1})	0.659 ± 0.017	0.530 ± 0.014	0.538 ± 0.025	0.421 ± 0.017

Values are triplicate reactor averages for the duration of the steady state period.

Table S2. *M. parvus* OBBP Reactor Data for Steady State Periods.

Retention Time (days)	2.8 ± 0.1	4.3 ± 0.2	5.8 ± 0.2	9.0 ± 0.4
Steady state period (days)	9–19	9–19	25–36	29–43
CH_4 (mg L^{-1})	1.34 ± 0.28	2.16 ± 0.47	0.42 ± 0.07	1.70 ± 0.50
O_2 (mg L^{-1})	4.19 ± 0.50	4.56 ± 0.49	5.41 ± 0.40	4.68 ± 0.85
Biomass (mg cell (dry weight) L^{-1})	112.85 ± 3.63	142.15 ± 5.21	162.72 ± 6.42	212.43 ± 8.25
CH_4 oxidation rate (mg CH_4 mL^{-1} day^{-1})	0.099 ± 0.002	0.095 ± 0.003	0.104 ± 0.001	0.097 ± 0.002
CH_4 oxidation rate (mg cell (dry weight) day^{-1})	0.878 ± 0.030	0.668 ± 0.024	0.641 ± 0.025	0.457 ± 0.022

Values are triplicate reactor averages for the duration of the steady state period.

Table S3. *M. album* BG8 Reactors Log2Fold Change in per cell *pmoA* transcript amounts during Substrate Limitation and Recovery Periods compared to Steady State.

		pmoA mRNA $cell^{-1}$ amounts log_2 Fold Change [a]	
Condition	Time (hours)	2.8-day retention time CH_4 off	4.3-day retention time O_2 off
Substrate limitation	0.5	-3.32	-4.26
	1.0	-7.28	-4.48
	2.1	-7.99	-4.69
	7.9	-8.21	-5.18
	12.0	-6.24	-4.66
	24.5	-5.59	-5.63
Recovery	8.1	-2.69	-3.51
	23.0	-0.83	-1.45

[a]Substrate limitation and recovery *pmoA* mRNA $cell^{-1}$ amounts compared to average *pmoA* mRNA $cell^{-1}$ amounts from preceding respective steady state condition samples.

Table S4. Mann–Whitney *U* Test comparisons between per cell *pmoA* transcript amounts during steady state, substrate limitation and recovery conditions for *M. album* BG8 reactors.

Condition	*p*-value[a]	
	2.8-day RT CH_4 off	4.3-day RT O_2 off
Steady state *vs*. starvation	$p < 0.001$	$p < 0.001$
Starvation *vs*. recovery	$p < 0.01$	$p < 0.05$
Steady state *vs*. recovery	$p = 0.14$	$p < 0.05$

[a]Evaluated on per cell *pmoA* transcript amounts determined using qPCR and RT-qPCR.

Table S5. General properties of *M. album* BG8 Transcriptome Samples.

Conditions	Steady State (2.8-day RT)		CH_4 off (2.8-day RT)		Steady State (4.3-day RT)		O_2 off (4.3-day RT)	
Reads ($\times 10^6$)	17.9	28.0	9.1	3.7	6.4	14.9	20.1	70.2
Reads aligned ($\times 10^6$)	13.4	19.2	6.4	3.1	4.3	10.8	13.4	48.0

Values presented for each condition correspond to biological replicates.

Table S6. Significant Differential gene expressions (DGE) for comparisons between CH_4 off, O_2 off steady state conditions in *M. album* BG8.

Test Condition	CH_4 off (2.8-day RT)	O_2 off (4.3-day RT)	Steady State (2.8-day RT)
Reference Condition	Steady State (2.8-day RT)	Steady State (4.3-day RT)	Steady State (4.3-day RT)
Upregulated	311	104	0
Downregulated	133	178	4
Total DGE[a]	444	282	4

[a]Significance was determined by an absolute $\log_2$fold change > | 1.0 |, and adjusted *p*-value < 0.05.

Table S7. Abbreviations of Compounds and Intermediates from Figure 6.

Abbreviation	Compound or Intermediate	Abbreviation	Compound or Intermediate
THF	tetrahydrofolate	GLT	glycerate
MFR	methanofuran	GLX	glyoxylate
H_4MPT	tetrahydromethanopterin	GLY	glycine
1,3DPG	1,3-bisphosphoglycerate	H6P	hexulose 6-phosphate
2PG	2-phosphoglycerate	HPYR	hydroxypyruvate
3PG	3-phosphoglycerate	ISOC	Isocitrate
6PG	6-phosphogluconate	KDPG	2-keto-3-deoxy-6-phosphogluconate
6PGL	6-phosphogluconolactonase	MAL	malate
AcCoA	acetyl-CoA	OAA	oxaloacetate
$CH{=}H_4MPT$	5,10-methenyl-H_4MPT	PEP	phosphoenolpyruvate
CH=THF	5,10-methenylTHF	PYR	pyruvate
CH_2-H_4MPT	5,10-methylene-H_4MPT	R5P	ribose 5-phosphate
CH_2-THF	5,10-MethyleneTHF	Ru5P	ribulose 5-phosphate
DHAP	dihydroxyacetone phosphate	S7P	sedoheptulose 7-phosphate
E4P	erythrose 4-phosphate	SER	serine
F6P	fructose 6-phosphate	SUC	succinate
FBP	fructose 1,6-bisphosphate	SuCoA	succinyl-CoA
FUM	fumarate	Tar-S	tartronate-S
G6P	glucose 6-phosphate	X5P	xylulose 5-phosphate
GAP	glyceraldehyde 3-phosphate	α-KG	α-ketoglutarate

REFERENCES

1. Tentori EF, Richardson RE. 2020. Methane Monooxygenase Gene Transcripts as Quantitative Biomarkers of Methanotrophic Activity in *Methylosinus trichosporium* OB3b. Appl Environ Microbiol 86:e01048-20.
